# Massive Endoscopic Screening for Esophageal and Gastric Cancers in a High-Risk Area of China

**DOI:** 10.1371/journal.pone.0145097

**Published:** 2015-12-23

**Authors:** Xianzhi Zheng, Xuhua Mao, Kun Xu, Lingshuang Lü, Xianzhen Peng, Min Wang, Guisheng Xu, Zhaolai Hua, Jianping Wang, Hengchuan Xue, Jianming Wang, Cheng Lu

**Affiliations:** 1 Department of Epidemiology, School of Public Health, Nanjing Medical University, Nanjing, 211166, China; 2 Department of Clinical Laboratory, Yixing People’s Hospital, Wuxi, 214200, China; 3 Department of Social Medicine and Health Education, School of Public Health, Nanjing Medical University, Nanjing, 211166, China; 4 Yangzhong Cancer Research Institute, Yangzhong People’s Hospital, Yangzhong, 212200, China; 5 The Innovation Center for Social Risk Governance in Health, School of Public Health, Nanjing Medical University, Nanjing, 211166, China; 6 Department of Breast, Nanjing Maternity and Child Health Hospital of Nanjing Medical University, Nanjing, 210004, China; University Hospital Llandough, UNITED KINGDOM

## Abstract

**Objective:**

This study aims to describe the findings from a massive endoscopic screening program in a high-risk area of China and to evaluate the prognosis of patients diagnosed through endoscopic screening compared with those diagnosed at usual hospital visits because of illness.

**Methods:**

In 2006, an early detection and treatment program was initiated in Yangzhong county, China. Local residents aged 40–69 years were eligible for free endoscopic screening. Endoscopic examination was performed with Lugol’s iodine staining, followed by biopsies. Patients diagnosed with esophageal or gastric cancer were referred for treatment and followed to assess their long-term survival status.

**Results:**

From 2006 through 2012, we screened 12453 participants, including 5334 (42.8%) men and 7119 (57.2%) women. The average age was 52.8±8.0 years. We detected 166 patients with upper digestive tract cancers, including 106 cancers in the esophagus (detection rate: 0.85%) and 60 cancers in the stomach (detection rate: 0.48%). Of these patients, 98.11% with esophageal cancer and 100% with gastric cancer were defined as at the early stage. In the process of follow-up, 17 patients died from cancer-related causes, and the median survival time was greater than 85 months. The overall survival rates for 1, 3 and 5 years were 98.0%, 90.0% and 89.0%, respectively. A significant positive effect was observed for the long-term survival of patients diagnosed through massive endoscopic screening.

**Conclusions:**

In a high-risk population, massive endoscopic screening can identify early stage carcinoma of esophageal and gastric cancers and improve patients’ prognosis through early detection and treatment.

## Introduction

Gastric cancer and esophageal cancer are two of the most common digestive tract cancers worldwide, accounting for approximate 8% and 4% of all new cancer cases, respectively [1]. During the last several decades, the morbidity and mortality of upper digestive tract cancers have significantly declined in the European countries [2, 3]; however, the disease burden of these cancers remains high in Eastern Asia [4]. Although an overall decrease in incident esophageal and gastric cancers has been observed in China, the decline has been relatively slower than that in other countries, and there has even been an increase in some specific regions [4].

Both esophageal cancer and gastric cancer are often asymptomatic in the early stages. When symptoms appear, the cancer has typically reached late stages [5]. The majority of patients show distant metastasis at the time of diagnosis, leading to a poor prognosis and lower survival rate [6]. The effectiveness of screening programs was confirmed in endemic regions [5]. The detection rate of endoscopy has been shown to be significantly higher than that of direct or indirect X-ray examination [7]. However, in most Asian countries, services for the early detection of cancer are limited. Health organizations and governments of countries with a high burden of cancers need to implement simple and economical screening tests, recognize risk groups and detect cancer patients at an early stage.

Although endoscopy is widely available in major cities in China, availability and accessibility in rural areas are limited. The cost of upper gastrointestinal endoscopy is relatively low, but less-privileged citizens might not be able to afford this procedure [7]. In 2004, an early detection and treatment program was initiated in China with special funds from the Ministry of Health. This program focused on screening for breast cancer, cervical cancer and digestive system cancers in areas with a heavy burden of disease [8]. As a pilot rural area, Yangzhong county was selected for the implementation of a population-based endoscopic screening program for the early detection and treatment of esophageal and gastric cancers. By 2012, more than 12453 individuals aged 40–69 years had participated in this project. What is the detection rate of precancerous lesions and carcinoma? How many patients have been detected at an early stage of cancer? Can endoscopic screening improve patients’ prognosis? Should we use the same screening strategy in both developed and developing high-risk areas of China? These key public health questions remain to be answered.

This study aims to describe the findings from an endoscopic screening program for upper gastrointestinal cancers in a high-risk area of China and to evaluate the prognosis of patients diagnosed through this screening program compared with those diagnosed through usual hospital visits because of illness.

## Methods

### Study site and subjects

Yangzhong is an island located in the middle of the Yangtze River in the southeastern part of Jiangsu province in China. It had a population of approximately 280,000 in 2013. Yangzhong is an area with high morbidity and mortality of both esophageal and gastric cancers [9]. To detect cancer early and diagnose and treat cancer patients, an endoscopic screening program was initiated in Yangzhong in 2006. Local residents aged 40–69 years were eligible for a free endoscopic screening. Those who voluntarily and willingly complied with the medical requirements were subject to the endoscopic examination. People with gastroscopy contradictions were excluded. Prior to the screening program, a village doctor visited the target populations in their home. A detailed explanation of the study’s goals and methods was given to each target individual. Informed consent was obtained from participants prior to their enrollment. A specific code with a defined day to visit a specific health center for endoscopic examination was given to each participant.

### Endoscopic screening

Target individuals visited the People’s Hospital of Yangzhong on their appointment date. A questionnaire was completed by a trained doctor from Yangzhong Cancer Research Institute. We collected participants’ demographic characteristics, smoking and alcohol drinking behaviors, history of specific diseases and family history of cancers in first-degree relatives. Then, all participants underwent a physical examination. The goals of the program and the endoscopy procedure were again explained to each individual. Equipped with a small chip camera and a non-coaxial optic fiber system, the standard esophagogastroduodenoscopy (EGD) was operated by experienced endoscopists to carry white light down the oropharynx to examine different levels of the gastrointestinal mucosa in the Endoscopy Room. The endoscopic results were recorded in a prepared form for the esophagus, stomach and duodenum. Iodine (Lugol’s solution) staining was used to detect esophageal mucosal lesions. Normal mucosal lesions contain an abundant amount of glycogen, which the iodine stains brown. Abnormal mucosal lesions such as squamous dysplasia and carcinoma in situ remain unstained due to the low glycogen content. Participants underwent biopsies for histopathologic evaluations at the designated sites of the esophagus and stomach according to the guidelines. Patients who were diagnosed through the endoscopic screening were referred to the department of surgery for timely treatment.

### Category of esophageal and gastric lesions

For the esophagus, severe hyperplasia /carcinoma in situ, mucosal carcinoma and submucosal carcinoma were categorized as the early stage cancer [10], and invasive carcinoma was categorized as the late stage cancer. For the stomach, mucosal high-grade tumors, mucosal carcinoma and submucosal carcinoma were categorized as the early stage cancer [11], and invasive gastric carcinoma was categorized as the late stage cancer.

### Survival analysis

Esophageal and gastric cancer patients diagnosed through the endoscopic screening program were followed to assess their treatment and long-term survival status. Information on the death of patients was routinely obtained from the death certificates in the vital statistical section of Yangzhong Center for Disease Control and Prevention (CDC). Through this approach, patients whose death information had not been received may be considered to be “alive” at that point in time. Furthermore, the mortality data were periodically matched with the database of incident cancers. Reports of death from hospitals, living status from the community, or loss to follow-up were updated until December 31, 2013. The censor was defined as patients being alive at the closing date or lost to follow-up or died from causes other than esophageal or gastric cancer. We further compared the survival rate of patients diagnosed through the endoscopic screening program and those diagnosed through usual hospital visits by frequency matching with age. Data of incident esophageal and gastric cancers and cancer-related death among the general population were derived from the Yangzhong Cancer Registry System. We identified the esophageal and gastric cancer cases using the 10th Revision of the International Classification of Diseases (code C15 and C16) [12].

### Data analysis

We used percentages to describe categorical data and means and standard deviations or medians and ranges to describe continuous data. The observed survival rates were calculated by the Kaplan-Meier method. The median survival time with 95% confidence intervals (CIs) was calculated to estimate patients’ survival status. The log-rank test was used to compare the survival time of the groups. The Cox proportional hazard regression model was used to explore survival-related factors. The level of significance was set as P < 0.05. All analyses were performed using SPSS 18.0 (IBM Corporation, New York, United States).

### Ethics consideration

This study was approved by the ethics committee of Nanjing Medical University. Written informed consent was obtained from all participants.

## Results

### Findings from the endoscopic screening

From 2006 through 2012, we screened 12453 participants, including 5334 (42.8%) men and 7119 (57.2%) women. The average age was 52.8±8.0 years (Table 1). The proportion of biopsy was over 99.3%. For esophagus, 2 (0.02%) subjects were diagnosed with invasive esophageal carcinoma, 7 (0.06%) subjects were diagnosed with submucosal esophageal carcinoma, 42 (0.34%) subjects were diagnosed with mucosal esophageal carcinoma, 55 (0.44%) subjects were diagnosed with severe hypoplasia/in situ, 143 (1.15%) subjects were diagnosed with moderate or mild hypoplasia and 925 (7.43%) subjects were diagnosed with esophageal mucosal inflammation. As shown in Table 2, the distribution of esophageal lesions was pyramid-shaped. With regard to the stomach, 13 (0.10%) subjects were diagnosed with submucosal gastric carcinoma, 34 (0.27%) subjects were diagnosed with mucosal gastric carcinoma, 13 (0.10%) subjects were diagnosed with high-grade intraepithelial neoplasia, 25 (0.20%) subjects were diagnosed with low-grade intraepithelial neoplasia, 3201 (25.70%) subjects were diagnosed with intestinal metaplasia, 10 (0.08%) subjects were diagnosed with atrophic gastritis and 9040 (72.59%) subjects were diagnosed with non-atrophic gastritis (Table 2).

**Table 1:** General information on study subjects participating in the screening program during the period of 2006–2012 in Yangzhong.

Year	N	Men	Women	Age (years)	
		n(%)	n(%)	Mean±SD	Median
2006	1006	399(39.7)	607(60.3)	52.7±8.1	52
2007	2163	929(42.9)	1234(57.1)	52.5±8.4	52
2008	875	365(41.7)	510(58.3)	52.9±8.1	53
2009	969	401(41.4)	568(58.6)	52.2±7.9	52
2010	3144	1390(44.2)	1754(55.8)	52.7±7.9	53
2011	1957	850(43.4)	1107(56.6)	52.7±7.9	53
2012	2339	1000(42.8)	1339(57.2)	53.3±7.8	53
Total	12453	5334(42.8)	7119(57.2)	52.8±8.0	53

**Table 2:** Endoscopic findings for the esophagus and stomach.

Lesions	N	%
**Esophagus**		
Invasive carcinoma	2	0.02
Submucosal carcinoma	7	0.06
Mucosal carcinoma	42	0.34
Severe hypoplasia/carcinoma in situ	55	0.44
Moderate hypoplasia	34	0.27
Mild hypoplasia	109	0.86
Esophageal mucosal inflammation	925	7.43
Normal	11279	90.57
**Stomach**		
Invasive carcinoma	0	0.00
Submucosal carcinoma	13	0.10
Mucosal carcinoma	34	0.27
High-grade intraepithelial neoplasia	13	0.10
Low-grade intraepithelial neoplasia	25	0.20
Intestinal metaplasia	3201	25.70
Atrophic gastritis	10	0.08
Non-atrophic gastritis	9040	72.59
Normal	117	0.94

### Cancer detection by gender and age

Through the massive endoscopic screening program, we detected 166 patients with upper digestive tract cancers, including 106 cancers in the esophagus (detection rate: 0.85%) and 60 cancers in stomach (detection rate: 0.48%). Of these patients, 98.11% with esophageal cancer and 100% with gastric cancer were defined as at the early stage. As age increased, the cancer detection rate significantly increased (P for trend < 0.001). For example, in subjects aged 40–44 years, the detection rate was 0.20% for esophageal cancer and 0.04% for gastric cancer, while in subjects aged over 65 years, the rates increased to 1.77% and 1.49%, respectively (Table 3). A significant gender disparity was observed in the detection rate of esophageal cancer and gastric cancer (Table 3).

**Table 3:** Cancer detection rates stratified by age and sex.

Variables	Screened subjects	Esophageal cancer			Gastric cancer		
		N(%)	χ^2^	P	N(%)	χ^2^	P
**Age**			71.78	<0.001		44.61	<0.001
40-	2455	5(0.20)			1(0.04)		
45-	2441	4(0.16)			3(0.12)		
50-	2259	12(0.53)			10(0.44)		
55-	2363	29(1.22)			15(0.63)		
60-	1862	37(1.99)			15(0.63)		
65-	1073	19(1.77)			16(1.49)		
	P for trend < 0.001				P for trend < 0.001		
**Sex**			9.45	0.002		12.10	<0.001
Men	5334	61(1.14)			39(0.73)		
Women	7119	45(0.63)			21(0.29)		

### Survival analysis

We followed all cancer patients who were diagnosed through the endoscopic screening program. By December 31, 2013, 18 of the patients died from cancer-related causes, and the median survival time (MST) was greater than 85 months. The overall survival rates for 1 year, 3 years and 5 years were 98.0%, 90.0% and 89.0%, respectively. No significant difference in survival rate was found between men and women. The overall survival was also similar for patients with esophageal cancer and those with gastric cancer. The stage of cancer lesions at the time of diagnosis was related to the patient’s prognosis. As the stage of cancer increased, the risk of death significantly increased (Table 4).

**Table 4:** Survival analysis on cancer patients detected in the endoscopic screening program.

Factors	N	Median survival time (months)	Survival rate (%)			HR(95% CI)	P	aHR(95%CI)*	P
			1 year	3 years	5 years				
**Sex**									
Men	100	>85	98	89	88	1		1	
Women	66	>85	97	90	90	0.75(0.28–1.98)	0.556	0.66(0.24–1.84)	0.428
**Sites**									
Esophagus	106	>85	98	92	91	1		1	
Stomach	60	>85	97	85	85	1.50(0.59–3.81)	0.392	1.79(0.67–4.81)	0.246
**Stage**									
Early	164	>84	98	91	90	1		1	
Late	2	>85	100	0	0	20.9(4.59–95.24)	<0.001	31.6(6.10–163.4)	<0.001

* aHR: adjusted hazard ratio, adjusted for sex, sites and stage.

### Effect of screening on patients’ survival

**Fig 1:**
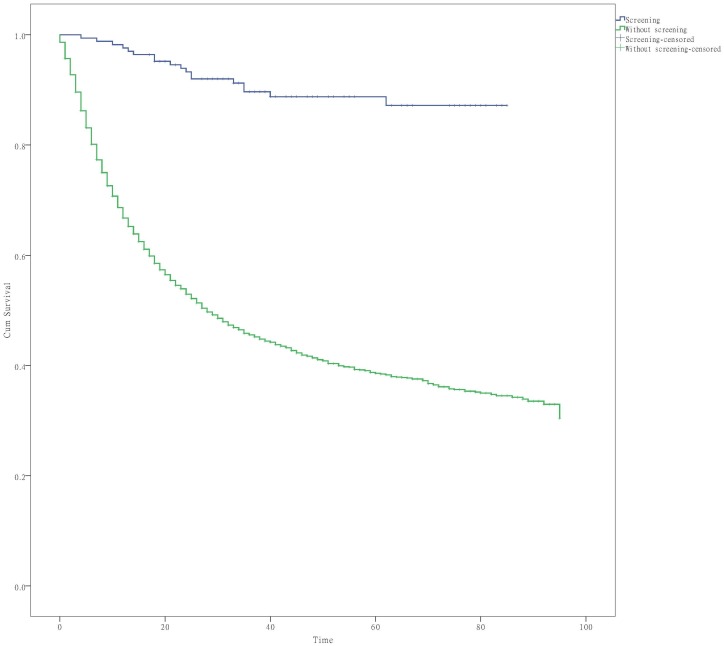
Survival curves of patients participating/not participating in the endoscopic screening program.

We further compared the survival of patients diagnosed in the endoscopic screening program and those diagnosed through regular hospital visits because of illness. A significantly positive effect was observed for the survival of patients participating in the massive endoscopic screening program (Log-rank test χ^2^ = 106.17, P < 0.001) (Fig 1).

## Discussion

Massive population-based esophageal and gastric cancer screenings of asymptomatic patients have been performed in Yangzhong county since 2006. This screening program identified patients with early upper digestive tract cancers who subsequently underwent esophagectomy or gastrostomy. We further conducted a survival analysis to clarify the role of endoscopic screening on patients’ prognosis. Our data showed that endoscopic screening in a high-risk population could identify potential invasive carcinoma, early stage carcinoma and precancerous lesions, improving prognosis through early detection and the treatment of esophageal and gastric carcinoma.

The burden of gastrointestinal cancer is increasing in Asia because of aging; rapid growth of the population; risk factors including tobacco smoking, obesity, malnutrition and the high prevalence of H. pylori infection [6]. However, in most Asian countries, cancer control programs or early detection and treatment services are limited despite this increase in disease. Cancer is one of the leading causes of death in urban and rural China [8]. Since 2005, a government-sponsored National Cancer Screening Program (NCSP) has been initiated in high-risk areas. This program mainly focuses on the early detection of cancers of the cervix, esophagus, colorectum, liver, nasopharynx, stomach and breast [13]. Previous studies have shown that survival rates could be increased through early detection and early treatment of esophageal and gastric cancer; however, there are few published studies evaluating the benefits of massive endoscopic screening. Thus, a systematic evaluation is necessary to provide a scientific basis for continuing and extending the program [14].

In the present study, we observed that the median survival time of screened patients was greater than 85 months and that the post-operational survival rates at 1, 3 and 5 years were 98.0%, 92.0% and 91.0%, respectively. These rates are better than those reported in previous studies [15, 16]. This result may be attributed to more patients being detected at an early stage of cancer in this study [17]. Our study demonstrated that cancer patients with endoscopic screening had significantly longer survival time than those without endoscopic screening. This result is in line with findings from other countries [18].

The early detection program for esophageal cancer in China began in the 1950s with a balloon cytology sampler [19]. However, the sensitivity for detecting esophageal cancer through biopsy was only 44% [20]. During the last decades, endoscopy has played a major role in the detection and characterization of neoplastic lesions along the digestive tract [21]. Detection of an abnormal mucosa in upper gastrointestinal endoscopy refers to the obvious elevation or depression, mucosal discoloration or interruption in the course of superficial capillaries [21]. However, the nonpolypoid lesions might be missed when the operator lacks cognitive knowledge and training.

Countries with high prevalence of esophageal and gastric cancers have initiated screening programs because early detection is associated with better outcomes [22–24]. In Korea, an endoscopic screening program was initiated in 1999 with financial support from the government. The screening involves upper endoscopy or upper gastrointestinal series (UGI) for patients 40 years or older every 2 years. As a result of this screening program, more than 50% of gastric cancers in Korea are diagnosed at an early stage, compared to fewer than 10% in Western countries [25, 26]. In 2005, a program named "Early Detection and Early Treatment of Esophageal and Cardiac Cancer" (EDETEC) was initiated in China. The cost-benefit analysis of screening for esophageal and cardiac cancers showed that the treatment cost savings were RMB 17730 and that the value of prolonged life was RMB 41214–137380, with the ratio of benefit-to-cost of 3.95–11.83 [27].

In the evaluation of the benefits of endoscopic screening, multiple interfering biases must be accounted. First, factors other than early screening and early-stage esophagectomy or gastrectomy may have contributed to the high survival rates observed in this study. Lead-time bias means that earlier diagnosis through screening will increase the measured survival time from diagnosis, regardless of whether screening helps people live to an older age [28]. Length bias is less intuitive. Because there is more time available to detect slow-growing rather than fast-growing tumors, routine screening is less likely to catch fast-growing, more lethal tumors. Patients with screen-detected tumors will inevitably have a longer survival time and a more favorable prognosis, even if screening has no real benefit [28]. Overdiagnosis can be regarded as the extreme case of length bias. Second, the stage migration bias refers to progress in the early detection of cancer occurring over time and independently from the screening intervention. Similarly, the progress in treatment improves the results and artificially increases the impact of the intervention on patients’ survival [21].

Certain limitations of our study merit further discussion. First, the long-term health economic effects of this program remain unknown. A cost-benefit analysis is needed to evaluate the feasibility of expanding the massive screening program. Second, a follow-up of precancerous lesions detected under endoscopy is warranted. Third, we screened the target population only one time. Whether repeated screening is necessary and what is an appropriate screening interval must be discussed in the future. Fourth, patients’ quality of life should be a subject of future research given the invasive nature of esophagectomy.

In conclusion, in a high-risk population of patients with upper digestive tract cancer, massive endoscopic screening can identify early stage carcinoma and improve patients’ prognosis through early detection and treatment. The survival of cases who received endoscopic screening was significantly better than that of age-matched controls diagnosed through regular hospital visits because of illness.
